# The Oncolytic Virus VSV-GP Is Effective against Malignant Melanoma

**DOI:** 10.3390/v10030108

**Published:** 2018-03-02

**Authors:** Janine Kimpel, Carles Urbiola, Iris Koske, Reinhard Tober, Zoltan Banki, Guido Wollmann, Dorothee von Laer

**Affiliations:** 1 Division of Virology, Medical University of Innsbruck, 6020 Innsbruck, Austria; Carles.Urbiola@i-med.ac.at (C.U.); Iris.Koske@i-med.ac.at (I.K.); reinhard.tober@i-med.ac.at (R.T.); Zoltan.banki@i-med.ac.at (Z.B.); guido.wollmann@i-med.ac.at (G.W.); dorothee.von-laer@i-med.ac.at (D.v.L.); 2 Christian Doppler Laboratory for Viral Immunotherapy of Cancer, 6020 Innsbruck, Austria; 3 Department of Neurosurgery, Yale University School of Medicine, New Haven, CT 06520, USA

**Keywords:** oncolytic virus, melanoma, vesicular stomatitis virus, VSV-GP

## Abstract

Previously, we described VSV-GP, a modified version of the vesicular stomatitis virus, as a non-neurotoxic oncolytic virus that is effective for the treatment of malignant glioblastoma and ovarian cancer. Here, we evaluate the therapeutic efficacy of VSV-GP for malignant melanoma. All of the human, mouse, and canine melanoma cell lines that were tested, alongside most primary human melanoma cultures, were infected by VSV-GP and efficiently killed. Additionally, we found that VSV-GP prolonged the survival of mice in both a xenograft and a syngeneic mouse model. However, only a few mice survived with long-term tumor remission. When we analyzed the factors that might limit VSV-GP’s efficacy, we found that vector-neutralizing antibodies did not play a role in this context, as even after eight subsequent immunizations and an observation time of 42 weeks, no vector-neutralizing antibodies were induced in VSV-GP immunized mice. In contrast, the type I IFN response might have contributed to the reduced efficacy of the therapy, as both of the cell lines that were used for the mouse models were able to mount a protective IFN response. Nevertheless, early treatment with VSV-GP also reduced the number and size of lung metastases in a syngeneic B16 mouse model. In summary, VSV-GP is a potent candidate for the treatment of malignant melanoma; however, factors limiting the efficacy of the virus need to be further explored.

## 1. Introduction

Melanoma is the leading cause (80%) of death from skin disease, although it accounts for only 4% of all dermatologic cancers [1]. It is the fifth most frequently diagnosed malignancy in men and the seventh in women, with an estimation of 91,270 new cases in the United States (US) in 2018 [2,3].

The overall five-year survival rate for melanoma is 91.3%, but the prognosis is only good for patients with early stage disease, where surgical removal is possible, and tumors have not yet spread. In contrast, the five-year survival rate for patients with late stage melanoma is much lower, e.g., only 16% for metastatic stage IV melanoma [4]. For this patient group, new immunotherapies have already considerably improved the prognosis. Monoclonal antibodies that block CTLA-4 or PD-1 act as checkpoint inhibitors and activate anti-tumoral T-cell responses [5,6]. However, only a fraction of tumor patients respond to checkpoint inhibitor therapy. A combination of two checkpoint inhibitors, e.g., ipilimumab and nivolumab, improves response rates. However, side effects are also enhanced, and still, a considerable proportion of patients does not respond [7,8].

To improve treatment efficacy, checkpoint inhibitors can be combined with other immunotherapies such as oncolytic viruses. At the end of 2015, the first oncolytic virus, the modified herpes simplex virus talimogene laherparepvec (T-VEC), was granted approval in the US and in the European Union (EU) for the treatment of advanced stage melanoma [9]. T-VEC expresses the immunostimulatory cytokine granulocyte–macrophage colony-stimulating factor (GM-CSF), and has led to the enhanced survival of patients with unresected stage IIIB to IV melanoma [10]. T-VEC is currently being tested in several clinical trials in combination with the checkpoint inhibitors ipilimumab or pembrolizumab (ClinicalTrials.gov identifier NCT03069378, NCT02626000, NCT02965716, NCT01740297, and NCT02263508). Initial small clinical studies indicate that T-VEC and ipilimumab [11,12], as well as T-VEC and pembrolizumab, act synergistically [13].

However, pre-existing or vector-induced antiviral immunity are expected to limit the efficacy of the oncolytic viruses that are currently in clinical development [14]. This is also true for the herpes simplex virus T-VEC, where pre-existing immunity even is a prerequisite for the safe application of the therapeutic dose. Virus-neutralizing antibodies are also expected to limit the systemic delivery of the virus.

We previously described a new oncolytic vesicular stomatitis virus, VSV-GP, in which the VSV glycoprotein G was substituted by the lymphocytic choriomeningitis virus (LCMV) glycoprotein GP. VSV-GP has several strengths, such as a fast replication cycle, no pre-existing immunity in the general population, and the capability to accommodate immunostimulatory cytokines or tumor antigens. We showed that systemic, intracranial, and intratumoral virus application is safe, even in immunodefective mice [15]. Further, VSV-GP lacks VSV’s inherent neurotoxicity, and does not readily induce neutralizing antibodies [16]. These features, and its favorable safety profile, make VSV-GP an ideal candidate for the treatment of advanced cancers [16,17,18].

We have previously shown in mouse models that intravenous treatment with VSV-GP is effective against subcutaneous as well as intracranial malignant glioblastoma [16]. As previously shown for the parental recombinant VSV, the oncolytic activity of VSV-GP depends on a defect type I IFN signaling in many tumors. Using interferon-competent ovarian cancer models, we could improve treatment efficacy by inhibiting the antiviral innate immune response with ruxolitinib, a Jak1/2 inhibitor [15].

Here, we analyze the efficacy of VSV-GP in cell lines and primary cultures as well as mouse models for malignant melanoma. We show that VSV-GP was not only effective in subcutaneous tumor models, it was also effective against developing lung metastases upon systemic treatment.

## 2. Materials and Methods

### 2.1. Ethics Statement

Animal experiments were performed in compliance with the national animal experimentation law (“Tierversuchsgesetz”), and animal trial permission was granted by national authorities (Bundesministerium für Wissenschaft und Forschung, #BMWF-66.011/0119-II/3b/2012 (31 August 2012) and BMWFW-66.011/0041-WF/V/3b/2016 (4 March 2016)).

### 2.2. Primary Cultures

Short-term cultures of human melanoma cells were a kind gift from Drs. R. Halaban and A. Bacchiocchi from the Specimen Resource Core of Yale University (SPORE) in Skin Cancer, and were designated with a YU prefix and an arbitrary letter code that was non-related to the patient's identity, in accordance with the Health Insurance Portability and Accountability Act (HIPAA) and the institutional Human Investigative Committee protocol. Melanoma tissue was derived from primary and metastatic sites with patients’ informed consent [19]. Cultures were maintained in Opti-MEM supplemented with 5% fetal calf serum (FCS). At 36 hours before virus inoculation, cells were seeded in 24-well plates. Medium was replaced at low volume with medium containing either VSV-GFP or VSV-GP-GFP for a final multiplicity of infection (MOI) of 0.1. At 24 h post-infection, cultures were analyzed for GFP expression and the presence of cytopathic effects using a fluorescence microscope. Ten microscopic fields were assessed per condition.

### 2.3. Cell Lines

A375 (from European Collection of Cell cultures) were maintained in Dulbecco’s modified medium (PAA) supplemented with 15% FCS (PAA), 4 mM l-Glutamine (Gibco, Waltham, MA, USA), 100 U/mL penicillin, and 100 mg/mL Streptomycin (Gibco). NW-1539 (kind gift from Prof. Dr. E. Jäger, II.Medizinische Klinik/Onkologie, Nordwestkrankenhaus Frankfurt/Main, Germany), B16-OVA (kind gift from Dr. Edith Lord and Dr. John Frelinger, University of Rochester Medical Center, USA) and MDA-MB-435 were maintained in Dulbeco’s modified medium supplemented with 10% FCS, 4 mM l-Glutamine, 100 U/mL penicillin, and 100 mg/mL Streptomycin. Mel-Juso and SK-MEL5 were maintained in RPMI-1640 medium (Gibco) supplemented with 10% FCS, 4 mM l-Glutamine, 100 U/mL penicillin, and 100 mg/mL Streptomycin. SK-MEL3 (from DSMZ) were maintained in McCoy’s A5 medium (Gibco) supplemented with 10% FCS, 100 U/mL penicillin, and 100 mg/mL Streptomycin. BHK-21 (ACCT) were maintained in GMEM (Gibco) supplemented with 10% FCS, 5% Tryptose (Gibco), 100 U/mL penicillin, and 100 mg/mL Streptomycin.

### 2.4. Virus Variants

VSV, VSV-GP, VSV-GFP, VSV-GP-GFP, VSV-GP-Luciferase, VSV*ΔG, and VSV*M_Q_ΔG (recombinant attenuated VSV with four mutations in the M protein and a deletion of the G protein), were described previously [15,16,18,20,21]. L929 or BHK-21 cells were used for the amplification of replication-competent VSV variants and BHK-21 or 293T cells expressing LCMV-GP or VSV-G for ΔG variants. Titers of replication-competent viruses were determined on BHK-21 cells, as described previously.

### 2.5. Tropism Assay

Cells were seeded in 24-well plates and were infected eight hours post-seeding with 10-fold serial dilutions of VSV*M_Q_ΔG-GP and VSV*M_Q_ΔG-G or mock-treated with phosphate buffered saline (PBS). After 16 hours, cells were analyzed via flow cytometry for GFP expression. The viral titer in each cell line was calculated and normalized to the obtained titer in the reference cell line BHK-21.

### 2.6. Killing Assay (WST-1 Assay)

Cells were seeded in 96-well plates. At 80% confluence, cells (12 technical replicates) were either infected with VSV wild-type or VSV-GP at an MOI of 0.1 or mock-treated with PBS. At indicated time points, 10 µL cell proliferation reagent WST-1 (Roche) were added per well. After four hours, color change was measured in a plate reader (Microplate reader Model 680, Biorad, Hercules, CA, USA) at 450 nm. For each well, a reference value, the wavelength at 650 nm, was subtracted. Obtained values were normalized to the mock-infected sample, and represented as a percentage of viable cells.

### 2.7. IFN Response

Cells were seeded in 96-well plates and pre-incubated overnight with the indicated concentrations of universal type-1 IFN (PBL, Piscataway, NJ, USA). The following morning, cells were infected with VSV wild-type or VSV-GP at an MOI of 0.1, 1, or 10. For each condition, quadruplicate samples were performed. As a positive, killing control cells were incubated, with a final concentration of 6.67 mM of H_2_O_2_. Three days after infection, cells were analyzed for viability using an MTT (3-(4,5-dimethylthiazol-2-yl)-2,5-diphenyltetrazolium bromide)-based in vitro cytotoxicity assay (Sigma-Aldrich, Saint Louis, MI, USA), according to the manufacturer’s recommendations. Plates were measured at 550 nm, and blank values of wells without cells were subtracted. Values were normalized to mock-infected cells that were not pre-treated with interferon (IFN), and represented as a percentage of viable cells.

### 2.8. Neutralization Assay

Heat-inactivated mouse plasma or the LCMV-neutralizing antibody Wen4, was serially diluted 1:5 fold starting with a 1:10 dilution. Dilutions were pre-incubated with the single-cycle infectious VSV*ΔG-GP virus (VSV*ΔG produced on cells expressing the LCMV GP) for one hour on ice. Subsequently, BHK-21 cells were infected in triplicate samples with the mixtures. Cells were incubated overnight at 37 °C. Cells were analyzed via flow cytometry for GFP expression. The mean value of the 1:10 dilution of the naïve plasma was set to 1, and all of the other values were given relative to this.

### 2.9. Fluorescence-Activated Cell Scanning Analysis

Blood from treated animals was collected two days after the third treatment using EDTA-coated tubes. 20 µL of blood per tube was stained with an APC (allophycocyanin)-conjugated SIINFEKL-specific tetramer (MBL) and fluorescence-conjugated antibodies against CD3, CD8, CD43, and CD44. Erythrocytes were lysed using ACK (ammonium-chloride-potassium) buffer, and samples were measured using a fluorescence-activated cell scanning (FACS) Canto II. Samples were analyzed using FlowJo software (FlowJo, Franklin Lakes, NJ, USA).

### 2.10. Bioluminescence Imaging (BLI)

Bioluminescence imaging (BLI) was performed using the Lumina In Vivo Imaging System (IVIS Lumina, Perkin Elmer). Mice were injected intraperitoneally with 1.5 mg d-luciferin (Promega, Madison, WI, USA).

### 2.11. Xenograft Mouse Model

Bilateral tumors (5 × 10^6^ A375 cells/tumor in 50 µL PBS) were injected subcutaneously into the flanks of NOD.CB17-Prkdcscid/J mice (NOD/SCID mice). Tumor growth was measured every other day with a caliper. Tumor volume was calculated as length × width^2^ × 0.4. When one tumor reached 0.07 cm^3^, both tumors were treated with intratumoral injections of either 10^7^ PFU of VSV-GP in 30 µL PBS, or mock-treated with 30 µL of PBS. A second, identical round of treatment was given 10 days after the first treatment. Mice were sacrificed by cervical dislocation when moribund or when tumor size exceeded 0.8 cm³.

### 2.12. Syngeneic Subcutaneous Mouse Model

C57BL/6J mice (Janvier) were transplanted subcutaneously (s.c.) in the right flank with 5 × 10^5^ murine B16-OVA melanoma cells in 100 µL PBS under isoflurane anesthesia. Tumor growth was monitored with a caliper starting three to five days post-injection, when the first tumors were palpable and visible. Tumor volumes were calculated as length × width^2^ × 0.4. Mice were sacrificed by cervical dislocation when either moribund, when tumor size exceeded 0.8 cm³, or when tumors ulcerated. Mice were divided into groups with similar average tumor volumes (*n* = 12 mice per group) on day 9 post-tumor cell transplantation. Mice were treated on days 9, 13, and 17 after tumor cell transplantation with either a low (2.36 × 10^4^ PFU), medium (4.72 × 10^5^ PFU), or high (2.36 × 10^7^ PFU) dose of VSV-GP-GFP intratumorally, or a high (2.36 × 10^7^ PFU) dose of VSV-GP-GFP intravenously. Virus was administered intratumorally in a volume of 30 µL PBS and intravenously in 100 µL PBS. PBS control mice were divided into two groups of six mice each, and one group was treated on days 9, 13, and 17 with 30 µL of PBS intratumorally, and the other with 100 µL of PBS intravenously.

### 2.13. Syngeneic Lung Metastasis Model

Murine melanoma lung metastases were established in C57BL/6 mice by intravenous injection of 1 × 10^6^ B16-OVA cells on day 0. Mice were treated intravenously with 5 × 10^8^ PFU VSV-GP on days 2, 4, 6, 8, and 10, or left untreated. On day 14, mice were sacrificed, and lungs were collected and stored in 1.5% formaldehyde. Lungs were dissected into individual lobes, and the number of visible metastases per lung was counted using a stereomicroscope.

### 2.14. Statistical Analysis

Statistical analysis was performed using GraphPad prism software (version 5, GraphPad Software, Inc., La Jolla, CA, USA), as indicated in the figure legends.

## 3. Results

### 3.1. Malignant Melanoma Cells Are Efficiently Lysed by VSV-GP In Vitro

**Figure 1:**
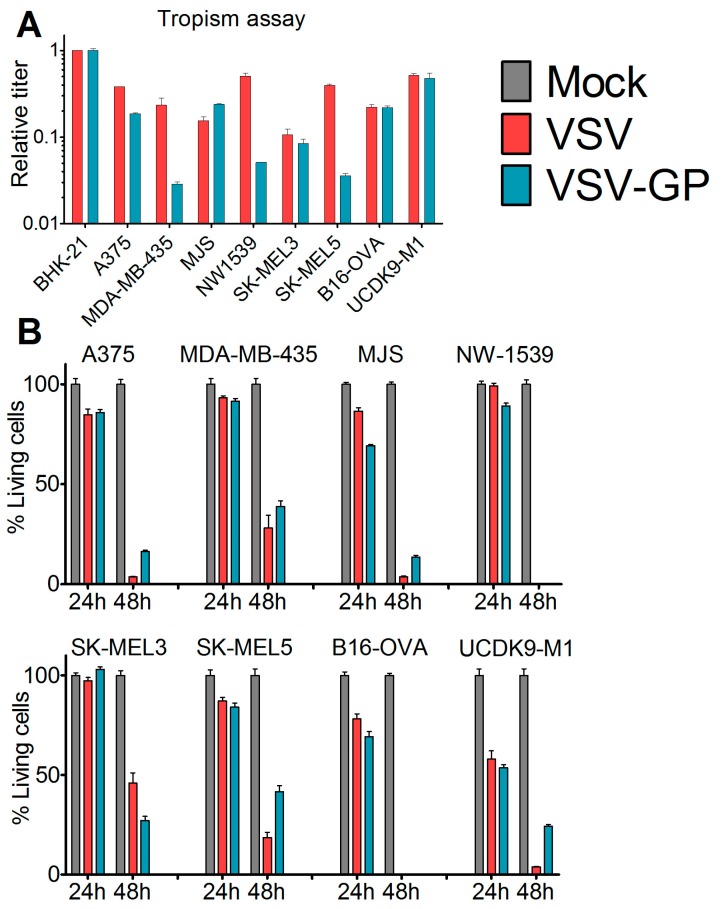

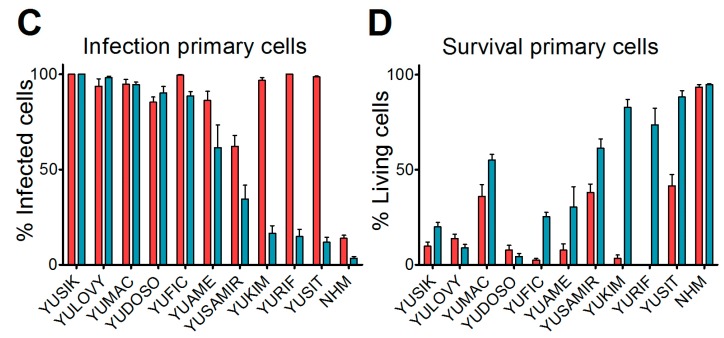
Melanoma cell lines and primary tumor cultures were efficiently infected and killed by VSV-GP. (**A**) The tropisms of VSV-GP and VSV wild-type for several human (A375, MDA-mB-435, MJS, NW1539, SK-MEL3, SK-MEL5) melanoma cell lines, one mouse (B16-OVA) melanoma cell line, and one dog (UCDK9-M1) melanoma cell line were analyzed. Cells were seeded as monolayers, and infected with 10-fold serial dilutions of the single cycle infective VSV*MQΔG-GP or VSV*MQΔG-G virus. BHK-21 cells were used as a reference. Sixteen hours post-infection, cells were analyzed for the percentage of GFP-positive cells via flow cytometry, and the titer for both viruses on each cell line was determined. The titers are given relative to the titer on BHK-21. Bars represent the means ± SEM of one representative of at least two independent experiments using duplicate or triplicate samples. For the control cell line, BHK-21, the mean and SEM of one representative experiment is shown; (**B**) monolayers of melanoma cell lines were infected with an MOI (multiplicity of infection) of 0.1 of VSV wild-type or VSV-GP in dodecaplicates. After 24 or 48 h, the viability of cells was determined using WST-1 assay. Values were normalized to the mock-infected sample, and represented as a percentage of surviving cells. Bars represent the mean ± SEM of one representative experiment of at least three independent experiments; (**C**,**D**) short-term cultures of human melanoma cells were seeded in 24-well plates, and 36 hours afterwards were infected with an MOI of 0.1 of VSV-GFP or VSV-GP-GFP. As control, normal melanocytes were used. Twenty-four hours post-infection, cells were analyzed in the fluorescence microscope for GFP positive, i.e., virus infected, (**C**) and living cells (**D**). Ten microscopic fields were assessed per condition. Bars represent mean ± SEM.

To assess the susceptibility of malignant melanoma to VSV-GP-mediated oncolytic virotherapy, we first analyzed a panel of human (A375, MDA-mB-435, MJS, NW1539, SK-MEL3, SK-MEL5), one mouse (B16-OVA), and one dog (UCDK9-M1) melanoma cell lines in vitro. Cells were infected with the single cycle infectious virus VSV*M_Q_ΔG, which lacks the glycoprotein gene and expresses GFP. During production, the VSV*M_Q_ΔG was transcomplemented with the glycoprotein of either VSV or LCMV. The percentage of infected cells was analyzed via GFP expression using flow cytometry and the titers for VSV-G or LCMV-GP pseudotyped viruses were calculated for each cell line. Titers were expressed relative to the reference cell line BHK-21 (Figure 1A). All of the cell lines could be infected with both viruses; however, for several cell lines, titers were slightly higher for the VSV-G pseudotype. We also analyzed the virus-induced killing of the melanoma cells after infection with replication-competent wild-type VSV or VSV-GP using the WST-1 assay (Figure 1B). Both viruses efficiently lysed all of the cell lines. It is of note that both of the cell lines that were used for the in vivo mouse models, A375 and B16-OVA, were efficiently lysed by VSV-GP.

We next analyzed short-term cultures of primary human malignant melanomas, which are considered more representative for cancer tissue. Cultures derived from 10 different patients [19] were infected with an MOI of 0.1 of either VSV-GFP or VSV-GP-GFP replication-competent viruses expressing GFP. In addition, normal human melanocytes were infected with both viruses. Twenty-four hours post-infection, cells were analyzed for GFP-positive infected cells and cytopathic effects using a fluorescence microscope. In the non-malignant, normal human melanocyte cultures, the majority of cells remained uninfected by both VSV-GFP and VSV-GP-GFP, and no cytopathic effects were observed. In contrast, all of the cultures of the primary malignant melanomas were efficiently infected by VSV-GFP, whereby VSV-GP-GFP showed lower levels of infection than VSV-GFP in five out of 10 cultures (Figure 1C). The productive infection of primary malignant melanoma cultures with VSV-GFP or VSV-GP-GFP, as determined by GFP expression in the cells, ultimately killed the cells (Figure 1D).

### 3.2. VSV-GP Prolongs Survival of Mice in Xenograft and Syngeneic Mouse Models

**Figure 2:**
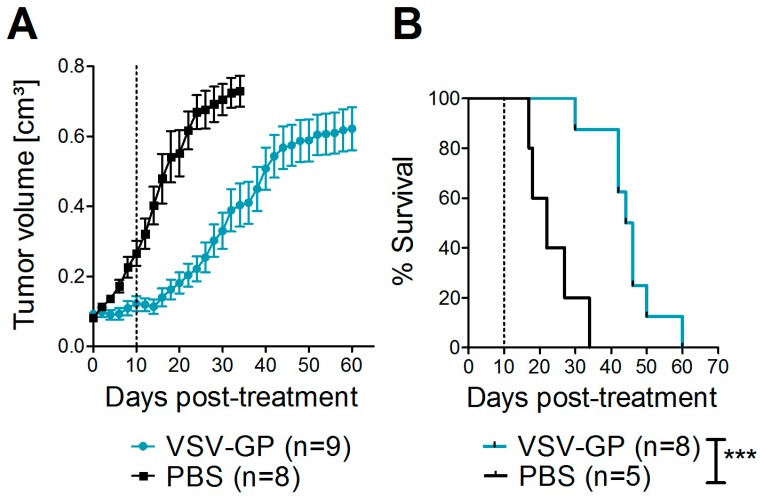
VSV-GP prolonged the survival of mice with human melanoma xenografts. A375 human melanoma cells were implanted subcutaneously into NOD/SCID (NOD.CB17-Prkdcscid/J) mice. Tumor growth was measured every two days using a caliper. When tumors reached 0.07 cm^3^ in volume, they were treated with either 10^7^ plaque forming units (PFU) of VSV-GP in 30 µL PBS, or mock-treated with 30 µL of PBS. A second round of treatment was given 10 days after the first treatment. Mice were euthanized when tumors reached a volume of 0.8 cm^3^. The mean ± SEM of tumor size (**A**) and % survival in a Kaplan–Meier curve (**B**) are shown. Dotted lines indicate the time point of the second treatment. *** *p* = 0.0004, Log-rank (Mantel–Cox) test.

Next, we asked whether these promising results in vitro could also be translated into efficacy of VSV-GP in melanoma mouse models. For a xenograft mouse model, human A375 malignant melanoma cells were transplanted into immune-deficient NOD/SCID mice. At a size of 0.07 cm³, tumors were either treated with PBS, or with two subsequent intratumoral doses of 10^7^ PFU VSV-GP. In the present study, we did not include an additional group of mice treated with wild-type VSV, as we saw massive neurotoxicity for these animals in previous studies with glioblastoma and ovarian cancer [15,16]. PBS-treated tumors rapidly grew, and all of the mice had to be sacrificed within 34 days after the start of therapy (Figure 2), while the growth of virus-treated tumors was significantly delayed. However, all of the tumors eventually grew. Median survival was significantly increased to 45 days in VSV-GP treated mice compared to 22 days for control mice (*** *p* = 0.0004, Log-rank (Mantel-Cox) Test).

**Figure 3:**
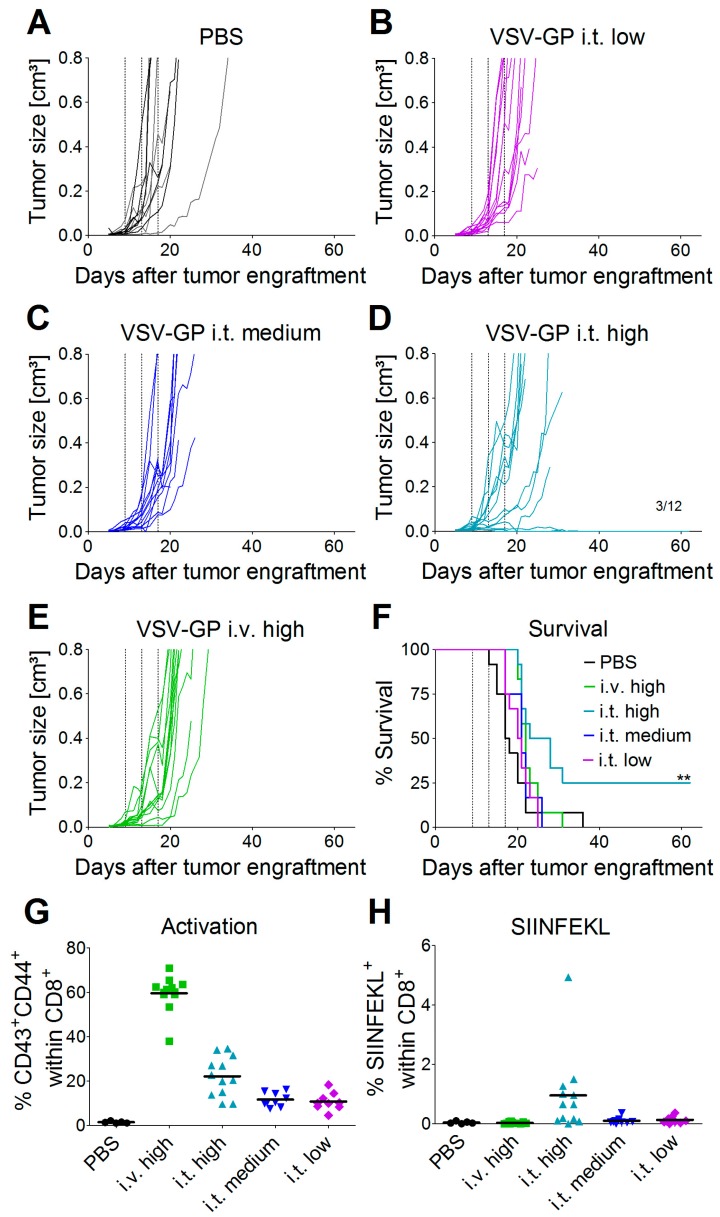
Efficacy of intratumoral treatment with VSV-GP in a syngeneic melanoma mouse model. 5 × 10^5^ B16-OVA cells were injected subcutaneously into C57BL/6 mice. On days 9, 13, and 17 post-transplantation, mice were treated with either PBS (six mice intratumorally = black lines, and six mice intravenously = grey lines, (**A**)), a low intratumoral (2.36 × 10^4^ PFU, (**B**)), a medium intratumoral (4.72 × 10^5^ PFU, (**C**)), a high intratumoral (2.36 × 10^7^ PFU, (**D**)) or a high intravenous (2.36 × 10^7^ PFU, (**E**)) dose of VSV-GP-GFP, *n* = 12. Animals were monitored for tumor growth, and sacrificed when tumor volume reached 0.8 cm³ or tumors ulcerated. (**F**) Kaplan–Meier survival curve. Dotted lines indicate time points of virus injection. ** *p* = 0.003, PBS vs. i.t. high, Log-rank (Mantel–Cox) Test. On day 2 after the third virus injection, blood was collected from the tail vein. The activation of CD8^+^ T-cells (**G**) and the percentage of SIINFEKL-specific cytotoxic T-cells (CTL) (**H**) were analyzed via flow cytometry.

Oncolytic viruses do not only directly lyse the tumor, they can also trigger a potent anti-tumor immune response. On the other hand, the antiviral immune response may limit viral replication and oncolysis. These aspects cannot be studied in xenograft models that rely on immune-deficient mice. To assess both oncolytic and immunologic effects, we tested VSV-GP in a syngeneic melanoma mouse model. C57BL/6 mice were transplanted subcutaneously with B16 cells expressing ovalbumin as a model tumor antigen. On day 9 post-tumor transplantation (average tumor size ~0.025 cm³), the treatment was started. Tumors were treated with three subsequent injections of a low (2.36 × 10^4^ PFU), medium (4.72 × 10^5^ PFU), or high (2.36 × 10^7^ PFU) intratumoral dose of VSV-GP-GFP, or a high (2.36 × 10^7^ PFU) dose of VSV-GP-GFP intravenously. Control tumors were treated with PBS only. Figure 3 shows the growth curves for individual tumors. All of the PBS-treated tumors rapidly grew, and mice had to be sacrificed within 36 days after the start of the treatment (Figure 3A,F and Figure S1). In contrast, animals treated intratumorally with VSV-GP-GFP responded to treatment with a delayed tumor growth or even tumor remission in a dose-dependent manner (Figure 3B–D and Figure S1). The survival of mice that were treated with the highest intratumoral dose of VSV-GP-GFP was significantly enhanced compared to control mice, with long-term remission in three out of 12 animals (Figure 3F). High-dose intravenous treatment (2.36 × 10^7^ PFU) was less efficient than high-dose intratumoral treatment (2.36 × 10^7^ PFU). Intravenously-treated tumors showed only a marginal growth delay, with the median survival of mice being prolonged to 22 days in virus-treated animals, compared with 17.5 days in the control group (Figure 3F). The median survival for the intratumorally-treated animals was 20.5 for the low dose, 21 days for the medium dose, and 25.5 days for the high dose. Only the high dose effect was statistically significant.

To study the immunologic effects of VSV-GP therapy, we analyzed immune responses in VSV-GP-GFP treated animals two days after the third treatment. We saw an increased activation of CD8^+^ T-cells in the blood of animals treated intratumorally, with the highest dose of VSV-GP-GFP compared to control animals (Figure 3G, (** *p* < 0.01, Kruskal–Wallis test with Dunn’s post-test). This systemic activation of CD8^+^ T-cells was significantly higher for animals that were treated intravenously rather than intratumorally (* *p* < 0.05, Kruskal–Wallis test with Dunn’s post-test). We also analyzed anti-tumoral T-cell responses against the model tumor antigen OVA. Seven out of 12 mice that were treated intratumorally with the high-dose of VSV-GP-GFP showed a significant proportion (>0.5% of CD8^+^ cells) of cytotoxic T-cells (CTL) directed against the ovalbumin (OVA) immunodominant epitope SIINFEKL (Figure 3H). The mean SIINFEKL-specific CTL response was significantly higher in the mice that were treated intratumorally compared with those that received intravenous treatment (* *p* < 0.05, Kruskal–Wallis test with Dunn’s post-test). It is interesting that the intravenously-treated mice showed no OVA-specific CTL response in the blood, although they had a higher general CTL activation compared with the intratumorally treated mice. These activated T-cells were most likely virus-specific.

As we saw an induction of CTL responses against the model tumor antigen OVA, we wanted to analyze whether this anti-tumoral immunity protects mice from rechallenge using the same tumor cells. B16-OVA cells were transplanted subcutaneously into C57BL/6 mice that showed a complete tumor remission after VSV-GP treatment. As a control, naïve C57BL/6 mice were also challenged with the same preparation of tumor cells. While the tumor in the naïve animals rapidly grew comparable to tumors in PBS-treated animals from Figure 3a, none of the VSV-GP treated animals with long-term tumor remission developed a tumor upon rechallenge within the 64-day observation period.

### 3.3. Type I IFN Induction But Not Neutralizing Antibodies Limit the Efficacy of VSV-GP

**Figure 4:**
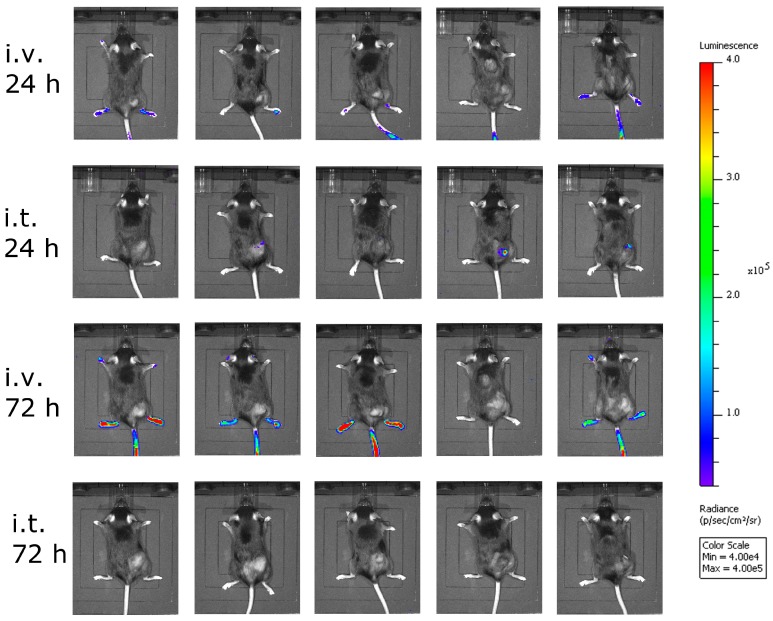
Less virus reached the tumor after intravenous virus application. B16-OVA cells were implanted subcutaneously into C57BL/6 mice. When tumors were palpable, mice were treated either intratumorally (i.t.) or intravenously (i.v.) with VSV-GP expressing luciferase. At 24 and 72 h post-treatment, mice were analyzed for luciferase activity using in vivo imaging. Five mice per group were analyzed. Color code depicts luminescence as photons/second/cm²/steradian [p/s/cm²/sr].

Next, we asked ourselves what limited the efficacy of VSV-GP in melanoma models. In a first experiment, we analyzed the amount of the virus that reached the tumor. Here, we used a virus containing luciferase as an additional transgene at position 5 in the viral genome between the *GP* and the *L* gene. We injected this virus intratumorally or intravenously into C57BL/6 mice carrying subcutaneous B16-OVA tumors, and analyzed the animals for luciferase activity using an in vivo imaging system (IVIS). Twenty-four hours after virus injection, no significant luciferase signal was seen in the intravenously-injected animals, while low levels of virus replication were detected in the animals that received the virus intratumorally (Figure 4). To answer the question how long virus replication proceeds, we analyzed the same animals again 72 hours after virus injection, and could hardly detect any virus within the tumor (Figure 4). Thus, the replication of VSV-GP in B16 melanomas was only transient.

**Figure 5:**
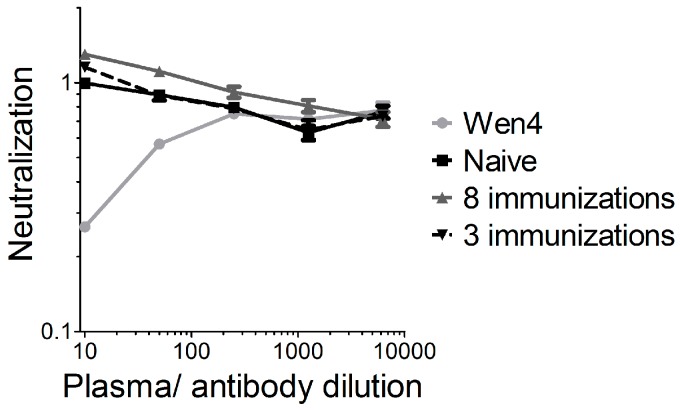
No vector-neutralizing antibodies were induced in mice after repeated VSV-GP injection. C57BL6 mice were immunized either three times (weeks 0, 8, and 16), and plasma was collected 23 weeks after the first immunization, or mice were immunized eight times within 40 weeks (weeks 0, 2, 4, 6, 8, 10, 14, and 40) and plasma was collected 42 weeks after the first immunization. Plasma pools from four vaccinated animals were pre-incubated with the replication incompetent VSV*ΔG-GP virus and subsequently BHK-21 were infected. As a negative control, plasma from a naïve mouse was analyzed. As a positive control, the lymphocytic choriomeningitis virus (LCMV)-neutralizing mouse monoclonal antibody Wen4 was used. The 1:10 dilution from the naïve mouse was set to 1. The graph shows the mean ± SEM of triplicate samples.

We previously showed that vector-neutralizing antibodies are not induced in mice after two intramuscular VSV-GP immunizations [17]. To further analyze this point, we immunized mice either three times (weeks 0, 8, and 16), and plasma was collected at week 23 after the first immunization, or mice were immunized eight times within 40 weeks (weeks 0, 2, 4, 6, 8, 10, 14, and 40), and plasma was collected 42 weeks after the first immunization. In a highly sensitive assay using a replication-defective VSVΔG virus pseudotyped with GP and expressing GFP, we detected no vector-neutralizing antibodies in both immunization schedules (Figure 5). The results from the xenograft model in immunodeficient mice also indicate that neutralizing antibodies are not responsible for the limited replication and efficacy of VSV-GP in melanoma.

**Figure 6:**
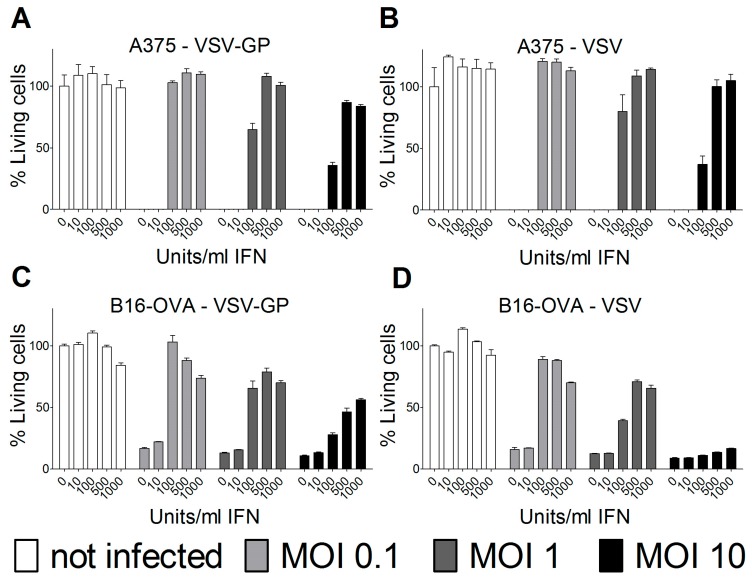
Type I interferon (IFN) limited VSV-GP replication in melanoma cell lines. Human A375 (**A**,**B**) or murine B16-OVA melanoma cells (**C**,**D**) were pre-incubated with indicated amounts of IFN for 16 hours. Subsequently, cells were infected in quadruplicates with VSV-GP or VSV wild-type at an MOI of 0.1, 1, or 10, or left uninfected as a negative control. Three days after infection, cells were analyzed for viability using an MTT assay. The viability of non-infected cells that were not pre-treated with IFN was set to 100%. The graph shows mean ± SEM of one representative experiment of at least two independent experiments.

In most human cancer cell lines, type I interferons (IFN) do not mount an antiviral innate immune response due to mutations in the IFN pathway. However, some human and several mouse cancer cell lines retain type I IFN responsiveness. For ovarian cancer and other tumor cell lines, we and others have found that an intact type I IFN response can limit the efficacy of oncolytic virus treatment. Therefore, we analyzed the IFN response in the melanoma cell lines used for the two mouse models. Both human A375 and mouse B16-OVA melanoma cell lines were protected by type I IFN from wild-type VSV and VSV-GP-mediated oncolysis (Figure 6). The protection could partially be overcome by high MOIs of virus in vitro. Thus, the interferon response in the tumor tissue most likely limited the replication and efficacy of VSV-GP in the two melanoma mouse models that were analyzed.

### 3.4. Systemic VSV-GP Application Effective Against Micro-Metastases

**Figure 7:**
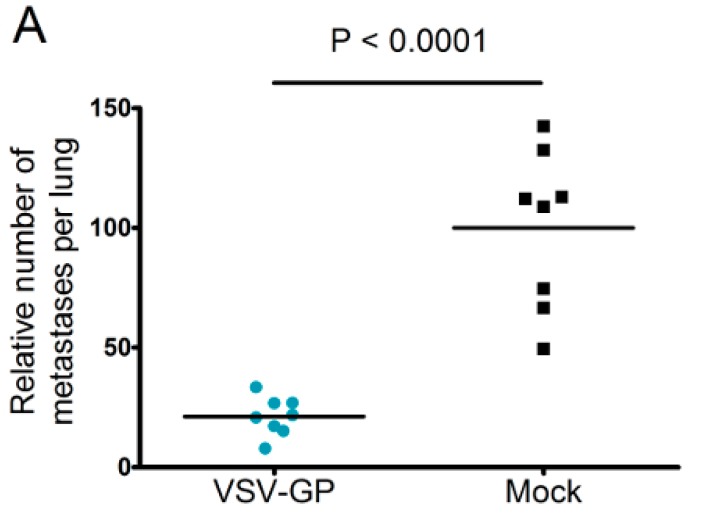

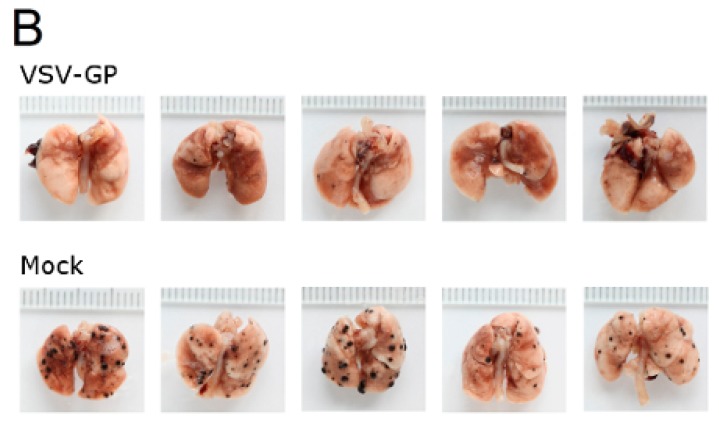
VSV-GP reduced the number of metastasis in a syngeneic mouse melanoma model. Murine melanoma lung metastases were established in C57BL/6 mice by the intravenous injection of 1 × 10^6^ B16-OVA cells. Mice were treated intravenously (i.v.) with 5 × 10^8^ PFU VSV-GP on days 2, 4, 6, 8, and 10, or were left untreated. On day 14, mice were sacrificed, and lungs were examined visually for metastases. The mean number of metastases for untreated animals was set to 100% (**A**). The graph shows data from two independent experiments, *n* = 8. The means and values for individual animals are depicted. *** *p* < 0.0001 (Unpaired, two-tailed *t*-test). Exemplary pictures from lungs from the second experiment are shown (**B**).

Neutralizing antibodies are expected to limit the efficacy of systemic oncolytic virus application, which is one reason why most oncolytic viruses are given intratumorally. However, as pre-existing antibodies against LCMV in the general population are rare, and VSV-GP treatment did not induce neutralizing antibodies in mice, we wanted to explore whether systemic treatment with VSV-GP inhibits metastases development. Lung micro-metastases were established in C57BL/6 mice by the intravenous injection of B16-OVA cells. Mice were treated intravenously every other day with five subsequent injections of 5 × 10^8^ PFU VSV-GP, or were left untreated. Mice were sacrificed on day 14 after tumor implantation. In treated animals, the number of lung metastasis was significantly reduced compared to untreated mice (Figure 7A). In addition, remaining metastases in the treated animals were of a smaller average size (Figure 7B).

## 4. Discussion

We previously described VSV-GP as a potent oncolytic virus for the treatment of glioblastoma and ovarian cancer [15,16]. Here, we explored malignant melanoma as a new target for VSV-GP oncolytic virotherapy. Advanced malignant melanomas often metastasize into the brain. In this setting, the lack of neurotropism/neurotoxicity, which is one of the strengths of VSV-GP compared to the parental VSV wild-type virus, is highly relevant. However, even in patients without brain metastases, the safety profile of a non-modified VSV will not be acceptable, and the virus will need to have further attenuations, e.g., the expression of IFNβ.

We found that in vitro, all of the melanoma cell lines from our panel, including a murine and a canine cell line, were susceptible to VSV-GP infection and VSV-GP-mediated cell killing. As established cell lines differ from primary tumors in many aspects, we also evaluated VSV-GP-mediated oncolysis in primary cultures of malignant melanoma. For the primary cultures, the situation was more diverse, as some samples were highly susceptible to VSV-GP infection and killing, whereas others seemed to be more resistant. Infection correlated well with the survival of cells. Cultures with a high infection rate were efficiently lysed by VSV-GP, whereas primary cultures with a low infection rate, such as YUKIM, YURIF, or YUSIT, showed a higher level of surviving cells one day after infection. It is of special interest that normal human melanocytes were not infected and lysed, neither by VSV-GP, nor by the parental VSV wild-type virus. This is in accordance with our previous data for ovarian cells, where benign immortalized human ovarian surface epithelial cells were not lysed by VSV-GP [15].

Despite the high susceptibility of melanoma cell lines and primary tumor cultures in vitro, we observed limitations of VSV-GP therapy in vivo in melanoma mouse models, especially when compared to the treatment efficacy in brain cancer [16]. Although in both melanoma models the tumor growth was delayed upon VSV-GP treatment, only a few animals with long-term tumor remission were observed. We and others have shown that type I interferon sensitivity of a tumor can limit efficacy of oncolytic virus treatment in mouse cancer models [15,22,23,24].

Therefore, we analyzed the IFN sensitivity of the two lines used for the mouse models and indeed found that both human A375 and mouse B16-OVA cells mounted a protective response against VSV and VSV-GP infection upon stimulation with universal IFN. This is in accordance with previous studies for these two cell lines [25,26]. Others have shown that A375 cells also produce high amounts of IFN in vitro [27]. This is especially important for the xenograft model, as the murine IFN produced by the tumor microenvironment will not sufficiently activate the signaling pathway in the human tumor cells to induce an antiviral state. In contrast, in the syngeneic B16-OVA mouse model, IFN produced by the tumor stroma cells can also induce an antiviral state in the sensitive B16-OVA cells, and thus limit the virus replication independent of IFN production by the tumor cells themselves.

In study by Stojdl et al., more than 80% of human tumor cell lines from the NCI60 panel had defects in their IFN response, and were therefore highly susceptible to VSV mediated killing in vitro [28]. Therefore, the IFN responsiveness in human A375 melanoma cells is a less common feature, which we also previously described for ovarian cancer cell lines [15]. Despite an efficient lysis of the human A2780 ovarian cancer cells by VSV-GP, the in vitro efficacy in an in vivo mouse model is limited. Similar to the A375 melanoma cells, the ovarian cancer line A2780 has an intact IFN responsiveness and production. In the latter, we showed that efficacy in the mouse model can be improved by inhibiting the downstream signaling of the IFNα receptor by the JAK1/2-inhibitor ruxolitinib [15]. We propose that a similar approach might also enhance the efficacy of VSV-GP in melanoma mouse models.

Thus, especially for the A375 melanoma, the IFN response might have limited virus spread and long-term therapeutic efficacy. The IFN response most likely also limited VSV-GP spread in B16 tumors, but as this is an immunocompetent, syngeneic model, the situation is more complex. B16 cells have been described to be rather resistant to VSV-mediated oncolysis in in vivo mouse models. In the B16 tumor model, replication of the virus and direct oncolysis seem to be of minor importance, as replication-defective VSV is as effective as a replication-competent virus [29]. Stimulation of innate immune mechanisms and the induction of anti-tumoral CTLs are more crucial factors for tumor control [30]. Janelle et al. showed that VSV replicates in subcutaneous B16 tumors for only a few days after intratumoral injection [31]. We made similar observations for VSV-GP using a virus variant expressing luciferase. One day after intratumoral virus injection, we detected only a low level of luciferase expression in the tumor, which was gone three days after treatment. Despite this low virus replication, and presumably also a low level of direct oncolysis, we could detect tumor-specific, i.e., OVA-specific, CTLs in the blood of mice treated intratumorally with the highest dose of VSV-GP. Only in this group, long-term tumor free survival was observed. In addition, these animals were protected from a rechallenge with the same B16-OVA tumor cells, indicating a long-lasting protection that is most likely mediated primarily by OVA-specific CTLs. To enhance this immunologic effect, arming the virus with immunostimulatory cytokines or tumor antigens is an interesting approach [26].

Although intravenous treatment led to the highest general activation of CD8^+^ T-cells in the blood of the animals, tumor-specific CTLs were not detected. This might be explained by the low level of virus replication in tumor tissue, and thus the low oncolysis and tumor antigen release after intravenous virus application.

For oncolytic viruses, such as herpes virus, polio, adenovirus, or a non-neurotoxic attenuated VSV variant expressing IFNβ, which are currently in clinical or pre-clinical testing, neutralizing antibodies against the vector backbone are either present in the general population, or are induced readily after the first administration, which limits the efficacy of repeated administration. We previously showed for VSV-GP that vector-neutralizing antibodies are not induced after two immunizations in mice, and that therefore, the immune response against a foreign antigen can be boosted by homologous immunizations [17]. For LCMV, the donor of the glycoprotein in our chimeric VSV-GP, neutralizing antibodies are induced only after long-term chronic infection [32]. It is speculated that it is difficult to induce neutralizing antibodies against LCMV due to the high glycosylation of the glycoprotein. We therefore increased the observation time after the immunization, and also the number of immunizations. In addition, we used a highly sensitive assay for the detection of neutralizing antibodies. However, even after eight subsequent immunizations, we could not detect any VSV-GP-neutralizing antibodies. Therefore, neutralizing antibodies did not limit virus homing to the tumor or local virus replication. Here, in addition to the IFN response as discussed above, poor tumor vascularization, hypoxia, and high intratumoral pressure might have limited intratumoral virus spread [33]. Further studies will be required in order to define potential additional relevant viral restriction factors in the B16 model for VSV-GP.

Despite the limited virus replication in B16 tumor tissue, the intravenous treatment of developing B16 lung micro-metastases with VSV-GP was effective. Thus, VSV-GP could have an impact on the growth of the metastases, or the development of new ones. In the metastases model, we could not compare VSV-GP’s efficacy to wild-type VSV, as in a preceding experiment, two-thirds of the tumor-free animals that were treated intravenously with a single high dose (5 × 10^8^ PFU) of wild-type VSV succumbed from neurotoxicity [16]. However, it will be interesting to compare the efficacy of VSV-GP in future studies with the attenuated VSV variant VSV-IFNβ, which is currently being tested in clinical trials for different tumor entities (ClinicalTrials.gov Identifier: NCT02923466, NCT01628640).

Taken together, VSV-GP is a promising new treatment option for malignant melanoma. Factors restricting VSV-GP’s efficacy will need to be further explored prior to clinical development. Understanding these restrictions is the basis for defining predictive biomarkers that will be essential for the selection of a target patient population that will benefit most from oncolytic virotherapy.

## Supplementary Materials

The Supplementary Materials are available online at http://www.mdpi.com/1999-4915/10/3/108/s1.
