# Supplementary material for: The Oncolytic Virus VSV-GP Is Effective against Malignant Melanoma
Source: Viruses. 2018 Mar 2;10(3):108. doi: 10.3390/v10030108 (PMC5869501; doi:10.3390/v10030108)
Supplement: Supplementary file 1 [file viruses-10-00108-s001.docx]

**Supplemental material**


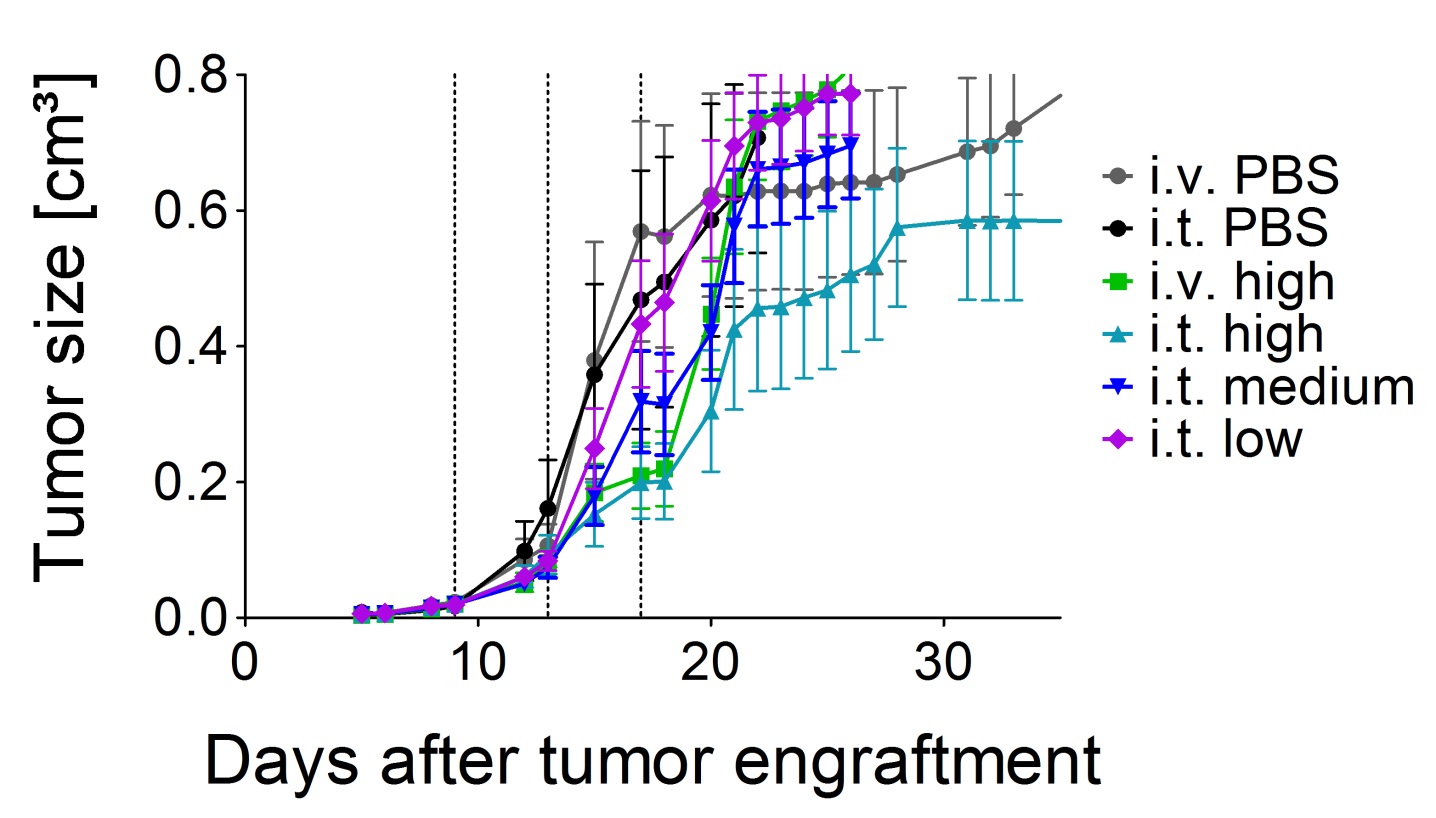


**Figure S1 Efficacy of intratumoral treatment with VSV-GP in a syngeneic melanoma mouse model.** 5x10^5^ B16-OVA cells were injected subcutaneously into C57BL/6 mice. On days 9, 13 and 17 post transplantation mice were treated with either PBS (6 mice intratumoraly = black line and 6 mice intravenously = grey line), a low intratumoral (2.36×104 PFU), a medium intratumoral (4.72×105 PFU), a high intratumoral (2.36×107 PFU) or a high intravenous (2.36×107 PFU) dose of VSV-GP-GFP, n = 12. Animals were monitored for tumor growth and sacrificed when tumor volume reached 0.8 cm³ or tumors ulcerated. Shown are mean ± SEM.
